# Step-Wise Management of Anemia in Patients With Chronic Kidney Disease in Primary Care: Qualitative Study

**DOI:** 10.1177/21501319221144955

**Published:** 2023-01-05

**Authors:** Gayathri Delanerolle, Anna Forbes, Jeremy van Vlymen, Hugh Gallagher, Nicholas Cole, Simon Hassan, Mohammad Tahir, Clare Bankhead, Tom Chan, Pauline A. Swift, Rebecca Suckling, Iain C. Macdougall, Mark Joy, Simon de Lusignan

**Affiliations:** 1 University of Oxford, Oxford, UK; 2 Epsom and St Helier University Hospitals NHS Trust, London, UK; 3 AT Medics, Edith Cavell Surgery, London, UK; 4 University of Surrey, Guildford, UK; 5 King’s College Hospital NHS Foundation Trust, London, UK

**Keywords:** anemia, chronic kidney disease, chronic renal insufficiency, medical record systems, computerized, general practice

## Abstract

**Introduction:**

Anemia is common in chronic kidney disease (CKD) and is associated with increased cardiovascular risk and reduced quality of life, but is often sub-optimally managed. Most patients are managed in primary care alongside other comorbidities. Interventions to improve the management of anemia in CKD in this setting are needed.

**Methods:**

We conducted a qualitative study to evaluate how an audit-based education (ABE) intervention might improve the management of anemia in CKD. We explored outcomes that would be relevant to practitioners and patients, that exposed variation of practice from National Institute for Health and Care Excellence (NICE) guidelines, and whether the intervention was feasible and acceptable.

**Results:**

Practitioners (n = 5 groups) and patients (n = 7) from 4 London general practices participated in discussions. Practitioners welcomed the evidence-based step-wise intervention. However, prescribing erythropoiesis-stimulating agents (ESAs) was felt to be outside of their scope of practice. There was a gap between NICE guidance and clinical practice in primary care. Iron studies were not well understood and anemia management was often conservative or delayed. Patients were often unaware of having CKD, and were more concerned about their other comorbidities, but largely trusted their GPs to manage them appropriately.

**Conclusions:**

The first steps of the intervention were welcomed by practitioners, but they expressed concerns about independently prescribing ESAs. Renal physicians and GPs could develop shared care protocols for ESA use in primary care. There is scope to improve awareness of renal anemia, and enhance knowledge of guideline recommendations; and our intervention should be modified accordingly.

## Introduction

Anemia is a common complication of chronic kidney disease (CKD) arising largely due to inadequate utilization of iron and erythropoietin production.^1^ It is a risk factor for cardiovascular morbidity, mortality, and reduced quality of life.^2^

In the United Kingdom CKD is largely managed in primary care and patients are often comorbid. The prevalence of anemia in CKD increases with declining kidney function, affecting 8.6% of people with stage 3-5 CKD.^3^ People living with diabetes develop anemia earlier in the course of kidney disease and are more likely to be anemic than those without diabetes.^4^ Anemia management in CKD has been extensively studied in specialist care settings and in those receiving dialysis.^5-7^ However, most people with CKD and anemia are managed in primary care.

Although the definition of anemia is not universally agreed, both NICE and the UK Kidney Association (UKKA) use hemoglobin of <110 g/L to define anemia in the context of CKD.^8,9^ The UKKA advise that CKD should be considered as a possible cause for anemia when eGFR <60 mL/min/1.73 m^2^. Guidelines recommend the investigation and treatment of other non-renal causes of anemia in the first instance. Once anemia has been established as being due to CKD, optimization of iron stores using oral or intravenous iron, and erythropoiesis-stimulating agents (ESAs) are advised. ESAs are effective at increasing hemoglobin in most patients, but there are concerns about their cardiovascular safety profile, particularly in patients with chronic inflammatory conditions and those who are poorly responsive to ESAs and requiring high doses. There may also be a role for less intensive and more holistic treatments, including effective medication management in primary care.

Despite guidelines on the management of anemia in CKD many people with anemia and CKD are sub-optimally managed. Data collected from 50 319 adults with CKD registered with practices who participated in the Quality Improvement in Chronic Kidney Disease (QICKD) trial identified several issues.^3^ Over three-quarters of people with anemia and CKD were prescribed 1 or more medications which may exacerbate anemia; nearly 3 quarters had been prescribed a non-steroidal anti-inflammatory drug, highlighting the need for more effective medication management. Oral iron was commonly prescribed; in over half (56.3%) of people with CKD and anemia and in two thirds (67.6%) of those with a low ferritin level, but it was often not effective at correcting anemia, consistent with the findings from previous studies.^10^

Despite these findings there have been few interventions targeted towards improving the management of anemia in CKD in a primary care setting. The aim of this study was to address this issue.

We conducted a qualitative study to explore the potential application of an audit-based education (ABE) intervention to improve the management of anemia in CKD in primary care. ABE is a non-judgmental peer led approach to service delivery improvement, which utilizes system data that is fed back from computerized medical record (CMR) about any gaps between current practice and national guidance. It has been successfully applied in a number of contexts, including the QICKD trial to improve blood pressure management in CKD.^11^ It takes advantage of the high quality of CMRs in UK primary care, which enables people with CKD to be identified and data about quality to be readily extracted. ABE meets the Medical Research Council’s definition of a complex intervention.

We explored whether ABE was an appropriate intervention to improve the management of anemia in CKD in primary care; what elements should be included in a step-wise approach and what differences between national guidance and adherence to that guidance would be most useful to feedback to practitioners.

## Methods

We assessed the feasibly of implementing a complex intervention to improve the management of anemia in people with CKD within primary care. It built on a similar exercise our group conducted prior to the QICKD trial. We conducted practitioner and patient-based exploration using focus groups and one-to-one interviews, respectively.

### Eligibility

The study was conducted using purposeful recruitment of 4 general practices based in South London using set inclusion criteria described below.

- Achievement of the pay-for-performance (P4P) for chronic disease management indicator score for CKD higher than the national average. The UK P4P scheme is called the Quality Outcomes Framework (QOF).
- At least the national average of people over 65 years (as CKD affects many older people).
- Membership of Oxford-Royal College of General Practitioners (RCGP) Research and Surveillance Centre (RSC) a nationally representative sentinel network of over 250 practices. This enables remote anonymous identifying of cases who meet the study criteria, and effective running of the subsequent feasibility study.

The recruitment of patients from the 4 general practices was based on the following eligibility criteria.

#### Inclusion criteria

- 18 years or older.
- Confirmed diagnosis of CKD stage 3 or 4 and/or eGFR of 15 to 59 ml/min/1.73 m^2^.
- Hemoglobin <110 g/L based on 2 readings taken between 7 and 30 days apart according to guideline recommendations.

#### Exclusion criteria

- Individuals who would not normally be directly managed by their GP, and hence unlikely to have their care influenced by any quality improvement intervention. This included people under secondary care for renal anemia, deemed frail, vulnerable, or living with dementia.
- Pre-existing conditions which might contribute to anemia. This included gastrointestinal tract hemorrhage, heart failure, and conditions causing malabsorption.

### Recruitment

We used a 3-stage process to identify patients who met the eligibility criteria outlined above. Firstly, we searched the CMR systems of the 4 participating general practices and generated a de-identified list of patients who met the inclusion criteria. A set of structured query language (SQL) searches were written and tested by the research team on a pseudonymised dataset held within the RSCs secure network. Secondly, using the set of tested queries, the investigating GP searched the information systems of the participating practices within the managed practice network and generated a de-identified list of patients who met the inclusion criteria. Finally, we constructed a special query to allow the patients who met the eligibility criteria to be identified by the lead study GP for each practice.

The lead GP in each practice accessed the individual’s complete CMR from the list of patients to determine if they were suitable for inclusion.

### Data collection

**Figure 1.**
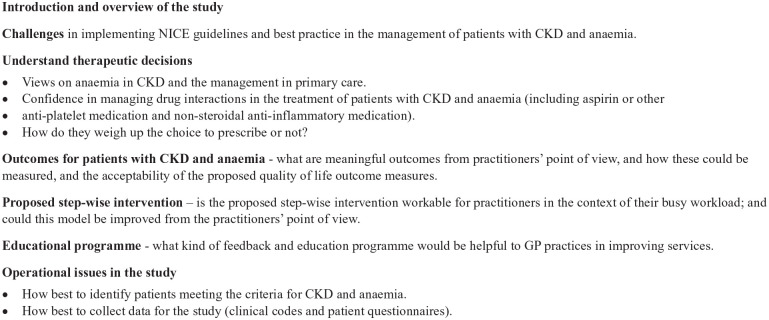
Topic guide for practitioner group discussions.

**Figure 2.**
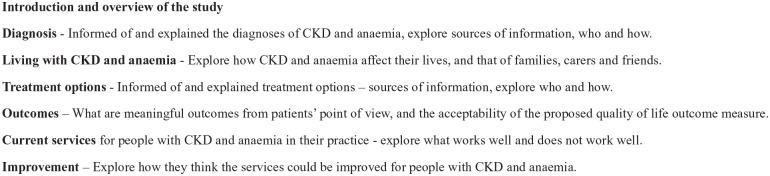
Topic guide for patient interviews.

**Figure 3.**
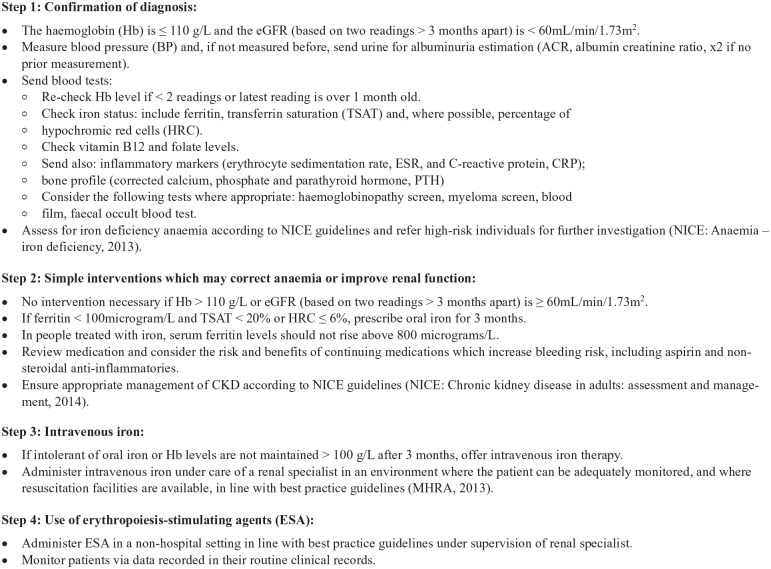
Proposed step-wise approach to the management of anemia in CKD.

Predefined topic guides, illustrated in Figures 1 and 2, were used to aid the discussion in the practitioner groups and patient interviews, and ensure qualitative data essential for meeting the study objectives were obtained. The proposed step-wise approach to the management of anemia in CKD that was presented to practitioners is detailed in Figure 3. With the explicit consent of the participants, the discussions were audio-recorded for analysis.

The conduct of the interviews and focus groups was not prescribed and was determined by the individual style of the facilitator/interviewer and the interactions they had with the participants to ensure a productive discussion that brought out the salient issues for the participants. A “vignette” of CKD patients with anemia was developed by the investigating GP, research team and renal physicians for the practitioners’ group discussions to add a sense of reality to the management of patients with CKD and their complex presentations in primary care.

Discussions were conducted in separate practitioner groups and patient groups to ensure that participants were able to speak freely: that practitioners could speak about strengths and limitations of their current practices and so that patients could express supportive or critical comments about current service provision in primary care.

The audio recordings were transcribed by a transcription service under a formal confidentiality agreement. Participants were identified only by pseudo-initials, to protect anonymity. The transcribed data files were imported into NVivo 10 (http://www.qsrinternational.com/nvivo/nvivo-products) for thematic analysis.

### Analysis

The coding schema was developed iteratively during the coding processes; the final themes and their interrelationships using an NVivo nodes tree.

## Results

We describe the profiles and report the themes that emerged from our discussions with practitioners and patients, separately.

### Practitioner Profiles

Five practitioner group discussions were conducted involving multidisciplinary healthcare staff. General practitioners and trainee doctors made up the largest group of participants of these discussions; however, some practice nurses and healthcare assistants also took part in these group discussions.

### Patient Profiles

Twenty patients agreed to participate in the interviews. However, a number of patients were not available at the time of interview. Seven patients took part in one-to-one interviews, including 3 males and 4 females with a mean age of 75 years old.

### Practitioner Themes

The practitioners’ perspective was that a step-wise model that converted national guidelines into pragmatic clinical decisions steps was useful and acceptable for the management of anemia in CKD, especially for busy clinical practitioners. One practitioner wished to start implementing the step-wise approach to the management of renal anemia straight away.

*Sounds really good. Could you email this (*the proposed step-wise model*) to XXX or do we have to wait?*

### Practitioner Themes; Diagnosis and Investigation of Anemia in CKD

Findings revealed that some practitioners used a lower threshold of hemoglobin level of 90 g/L or below, much lower than NICE recommended guidelines, before considering referral to secondary care services.

The discussion of the diagnosis and investigation of anemia in CKD revealed that practitioners agreed that it would be reasonable to take a full history, conduct an examination, and instigate a range of screening blood and urine investigations to establish the presence of anemia and to exclude any reversible causes of feeling of fatigue and anemia, such as malignancy or blood loss due to other causes. Typically:

*. . .essentially what I’m trying to make sure is that this anaemia is not down to any other cause, so looking at the mean cell volume, I’m looking at the platelet count in case there’s an inflammatory cause for it, I’m also looking to see if there’s a high eosinophil count in case this is down to some membrane issue within the kidneys. I’d then send off thyroid function to make sure the anaemia is not secondary to a hypothyroidism . . ., I want to check the iron levels to correspond with the anaemia in case this is an iron deficiency anaemia coming from another otherwise unknown cause such as a neoplastic lesion somewhere, or syndrome. . ..*

The use of fecal occult blood tests outside of bowel screening was not common practice at the time of the practitioner group discussion. The measurement of transferrin saturation and recognition of hypochromic cells were under appreciated as useful diagnostic tools to assess iron repletion, and a normal ferritin was often misinterpreted as meaning that iron stores were replete and no further intervention was necessary.

### Practitioner Themes; Management of Anemia in CKD

Once non renal causes of anemia had been ruled out, we found that the treatment for anemia would commonly be either conservative, delayed, or non-existent. Prescriptions of iron and referrals to renal specialists would be considered where anemia had been confirmed, but generally the clinical management tended to be confined to ongoing and regular monitoring, with more focus on kidney function than on anemia management. The following transcripts are illustrative of this point:

*I’d optimise her diet as well for iron but yeah, consider giving iron.*

*. . .Because I’d do monitoring. . . refer if it was less, if it was more than 9 but if their eGFR dropped then I’d refer more quickly I think.*

*. . .so well if it’d dropped below 9 then you’d refer, but say it’s dropped about, it depends how often the blood’s taken, every month, every six month, depending, so if it was 6 months and it’d dropped by 10 then yeah, dropped by 1 from 10 to 9 then yeah, I’d refer, or 11, 11 to 10, because that’s quite a significant drop, so over the 6 month period then I’d probably refer to renal in that case as well.*

The data suggested that practitioners positively welcomed an evidence-based approach to medical treatment. However, when the discussion turned to NICE guidelines, a number of the participants did not seem to be very familiar with latest NICE guidelines on anemia management in chronic kidney disease:

*I’m personally not familiar with them, I’m being really honest. . .’*

*‘I’m not familiar with it either.*

*. . .I can only bank on my experience in doing medicine before, any NICE guidelines that I’ve read are probably a couple of years historically now. . .*

An important finding was of some concerns about the unsupervised use of ESAs in primary care. These concerns would be alleviated by support and follow-up by specialists in the use of ESAs.

*. . . I don’t think the EPO should be kind of initiated in a community setting, I think it’s fine if they want us to give it, you know, to prescribe it or whatever in the community, but as long as they follow it up and change the doses, I think that’s fine. And the continuity, yes fine.*

### Patient Themes

All the patients had comorbidities other than CKD and anemia and often physical limitations to their activities of daily living. These additional long-term conditions and physical limitations included diabetes, cardiovascular disease, arthritis, aches and pains, and breathlessness. They often had symptoms that might have been made worse by their anemia, including shortness of breath.

The data indicated that a feeling of tiredness was a commonly cited health problem associated with renal anemia, which together with the effects of other health problems, further restricted their activities of daily living.

*. . .I’ve got severe heart disease, I’ve had a triple bypass, I’ve got type 2 diabetes, and I’ve got a defibrillator fitted, got a hernia with a stoma, I have got hardening of the arteries in the legs. . .*

*. . .get sort of really out of breath. . .*

*. . .Not very good, no, I’ve had a very bad bout of constipation, and an awful lot of pain from my groin to my knee on my right leg, and they say it’s a result of iron injections, and very strong painkillers, but apart from that I don’t know, I’ve just put it down to arthritis. . .*

For many, the family was the main source of support and practical help to their activities of daily living. The most commonly cited help provided by family members was help with personal care and shopping.

The interview data suggested that this sample of relatively older patients had confidence in their doctors and nurses, and that they tended to be deferential to their doctors. They seemed generally to be content to trust the judgment of their doctors and nurses in doing the best for them. Some felt their doctors already had to cope with other patients with more serious conditions and therefore felt reluctant to bother them.

*. . .Well I don’t see that it’s my place to say, I’m not knowledgeable enough to comment, you know?. . .*

*Well I don’t know much about medical things so I just take whatever the doctor or the nurses say to me, I don’t let that worry my brain because if I worry then it’ll get worse.*

*. . .But they’ve got enough problems with more serious cases than mine, so I try to keep away as much as I can. . .*

The patient interview data further suggested that some CKD patients in this sample were not aware of the availability of information on diagnosis, treatment and management. There were suggestions that where such information was given to patients, it typically derived from hospital out-patient clinics. The patients did not seem actively to seek further information on their condition from their general practitioners. Where they had been informed, they had a tendency to forget what had been explained to them in terms of diagnosis and treatment options.

*I can’t understand how my kidneys are suddenly. . . nobody tell me about that I’ve got. . . my kidneys don’t work until just last week someone ring from the clinic to say that . . . explained to me*

*. . .Not really, no, I can’t really say an awful lot ‘cos my memory’s not very good and they tell me something and in no time at all I can’t explain it to anybody else, I’ve forgotten it, you know, I can’t really recall that they’ve told me an awful lot though. . .*

The interview data suggested that there is generally a low expectation on the part of the patients of their treatment and the outcomes of their treatment. For some of these patients, it had been a long time since they had been in good health. The additional problems of chronic kidney disease did not seem to add much to their sense of ill health. Some patients seemed to “down-play” the impact of the symptoms of renal anemia, the effects and side effects of medications, and the need to make changes to their daily routine to attend additional investigations and appointments associated with treatment of their chronic kidney problems. Many had simply accepted their health problems, and the limitations placed on their lives as a result of their multiple health problems.

*. . .Well I don’t really know, it’s very difficult to say, I can’t say that I’ve felt, you know, in good health for such a long time now that any slight change I don’t notice any different really. . .*

*. . .Well I know I won’t be fighting fit but I’d like to feel a bit better sometimes but what it would entail I don’t know. . .*

*. . . I don’t know if something can be done, you know, I’m fine with it. . .*

### Other Findings

We demonstrated that we could successfully run complex searches of CMR to identify patients who met eligibility criteria for our planned feasibility study. However, these records still needed a manual review by the GP in the individual practice prior to invitation to interview.

Based on these findings, a step-wise ABE intervention for the management of anemia in CKD was developed.

## Discussion

### Principal Findings

Our diagnostic analysis suggested that practitioners would welcome the introduction of an evidence based structured approach to the management of anemia in CKD. We identified that renal anemia is under-recognized and sub-optimally management in primary care. Specifically, the interpretation of iron availability and stores was not well understood, and current clinical practice was often not in accordance with guideline recommendations.^8,9^ Anemia management was often conservative and hemoglobin thresholds used to trigger treatment or specialist referral were lower than those recommended in guidelines. The multi-morbidity of this population adds to the complexity. Finally, there were concerns over initiating ESAs in primary care due to a lack of clinical expertise and support from specialist care to safely and cost effectively deliver this treatment.

The patients in this study had strong, trusting relationships with their primary care physicians and deferred to their judgment in doing the best for them. There was generally a low expectation on the part of the patients as to their treatment or the outcomes of their treatment. Some patients expressed a wish to feel better without specifying what “feeling better” means. Many have accepted their health problems and come to terms with the limitations placed on their lives as a result of their comorbidities.

### Implications of the Findings

A step-wise approach to the management of anemia in CKD is feasible and welcomed in principle. However, there is a significant gap between national guidance and current clinical practice with regard to diagnosing and investigating anemia, as well as interpreting and responding to results in patients with CKD. The step-wise intervention needs to address this and consider a holistic approach given the age and multi-morbidity of this population.

The intervention also needs to consider the valid concerns about ESA initiation and prescribing in primary care. Shared care agreements, such as those utilized within rheumatology and oncology could be adopted in the management of anemia in CKD in primary care.

National targets and incentives could be used to optimize the adoption of the step-wise approach we have developed. For example, the Quality and Outcomes Framework which provides financial incentives to achieve targets by individual primary care providers; and an Investment and Impact Fund (IIF) provided to clusters of practices in groups called Primary Care Networks. The addition of anemia management in CKD to these incentive schemes should be considered.

### Comparison With the Literature

Anemia is a common complication of CKD, affecting 8.6% of patients with stage 3-5 CKD.^3^ It is consistently associated with increased mortality, cardiovascular events, and CKD progression, as evidenced by a recent systematic review.^2^ CKD is largely managed within primary care, highlighting the importance of effectively treating anemia in this setting. Despite this, few studies have explored interventions to improve the management of anemia in CKD in primary care.

A research group in New Zealand designed and implemented a program to identify and treat renal anemia in 79 patients with CKD stage 3 and 4 in primary care.^12^ A simple referral and management protocol for general practitioners was implemented, remotely supported by a nephrologist and nurse co-ordination team. This enabled general practitioners to safely use ESAs. They demonstrated a significant improvement in hemoglobin and found that treatment of anemia with ESAs can be successfully accomplished in a primary care setting by general practitioners, without the need for many to attend specialist nephrology clinics. However, this was a small study, they did not optimize iron status with parenteral iron therapy, evaluate hard clinical end-points or consider the cardiovascular safety of ESAs in this population.

Newer therapies such as Roxadustat, an oral hypoxia-inducible factor inhibitor that stimulates erythropoiesis, are emerging. NICE have recently published guidance recommending Roxadustat in the treatment of symptomatic anemia associated with CKD, and it is important that new treatments become embedded into routine clinical practice.^13^ Shared care agreements for ESAs also needs to be explored further with careful consideration of their cardiovascular safety.

### Limitations

The study was conducted in a small number of general practices in a specific geographical area. Consequently, the findings may reflect location-specific practice patterns and may not be representative of wider clinical practice. Although 20 patients agreed to participate interviews were conducted in only 7 patients, limiting the patient data available for thematic analysis.

We recruited general practices based on having at least the national average of people 65 years and older. However, given the increased prevalence of CKD in the elderly population it would have been better to focus on practices with a greater than average proportion of people 65 years and older.

## Conclusions

We have established that our planned step-wise intervention to improve the management of anemia in CKD would be welcome in primary care, and an ability to identify complex cases is feasible. CKD and anemia are usually part of comorbidity such that management is likely to be complex and holistic. It is our view that initiating ESAs in primary care would be best achieved with a national shared care plan. Our findings have provided important guidance about what elements should form part of our planned step-wise ABE intervention for anemia in CKD.

## References

[1] Portoles J Martin L Broseta JJ Cases A. Anemia in chronic kidney disease: from pathophysiology and current treatments, to future agents. Front Med (Lausanne). 2021;8:642296. doi:10.3389/fmed.2021.642296 33842503 PMC8032930

[2] Palaka E Grandy S van Haalen H McEwan P Darlington O. The impact of CKD anaemia on patients: incidence, risk factors, and clinical outcomes-a systematic literature review. Int J Nephrol. 2020;2020:7692376. doi:10.1155/2020/7692376 32665863 PMC7349626

[3] Dmitrieva O de Lusignan S Macdougall IC , et al. Association of anaemia in primary care patients with chronic kidney disease: cross sectional study of quality improvement in chronic kidney disease (QICKD) trial data. BMC Nephrol. 2013;14:24. doi:10.1186/1471-2369-14-24 23351270 PMC3626717

[4] Singh DK Winocour P Farrington K. Erythropoietic stress and anemia in diabetes mellitus. Nat Rev Endocrinol. 2009;5(4):204-10. doi:10.1038/nrendo.2009.17 19352318

[5] Macdougall IC White C Anker SD , et al. Intravenous iron in patients undergoing maintenance hemodialysis. N Engl J Med. 2019;380(5):447-458. doi:10.1056/NEJMoa1810742 30365356

[6] Cody J Daly C Campbell M , et al. Recombinant human erythropoietin for chronic renal failure anaemia in pre-dialysis patients. Cochrane Database Syst Rev. 2005;3:CD003266. doi:10.1002/14651858.CD003266.pub2 16034896

[7] Horl WH. Anaemia management and mortality risk in chronic kidney disease. Nat Rev Nephrol. 2013;9(5):291-301. doi:10.1038/nrneph.2013.21 23438972

[8] Padhi S Glen J Pordes BA Thomas ME Guideline Development G . Management of anaemia in chronic kidney disease: summary of updated NICE guidance. BMJ. 2015;350:h2258. doi:10.1136/bmj.h2258 26044132

[9] Mikhail A Brown C Williams JA , et al. Renal association clinical practice guideline on anaemia of chronic kidney disease. BMC Nephrol. 2017;18(1):345. doi:10.1186/s12882-017-0688-1 29191165 PMC5709852

[10] Shepshelovich D Rozen-Zvi B Avni T Gafter U Gafter-Gvili A. Intravenous versus oral iron supplementation for the treatment of anemia in CKD: an updated systematic review and meta-analysis. Am J Kidney Dis. 2016;68(5):677-690. doi:10.1053/j.ajkd.2016.04.018 27321965

[11] Lusignan S Gallagher H Jones S , et al. Audit-based education lowers systolic blood pressure in chronic kidney disease: the Quality Improvement in CKD (QICKD) trial results. Kidney Int. 2013;84(3):609-20. doi:10.1038/ki.2013.96 PMC3778715 23536132

[12] Madhan KK Chamberlain M Anderson E. Anaemia in patients with chronic kidney disease: management with epoetin beta in primary care setting in New Zealand. Nephrology (Carlton). 2008;13:428-32. doi:10.1111/j.1440-1797.2008.00935.x 18331435

[13] National Institute for Health Care and Excellence. Roxadustat for Treating Anaemia in People With Chronic Kidney Disease. Technology Appraisal Guidance [TA807]. 2022. Accessed September 8, 2022. https://www.nice.org.uk/guidance/ta807 40493731

